# Supplementary material for: Metaviromes Reveal the Dynamics of Pseudomonas Host-Specific Phages Cultured and Uncultured by Plaque Assay
Source: Viruses. 2021 May 21;13(6):959. doi: 10.3390/v13060959 (PMC8224292; doi:10.3390/v13060959)
Supplement: Supplementary file 1 [file viruses-13-00959-s001.zip › viruses-1217889-SI.pdf]

viruses

MDPI

Supplementary material for the article:

Metaviromes reveal the dynamics of *Pseudomonas* host-specific phages cultured and uncultured by plaque assay

Katrine Wacenius Skov Alanin[1,2,], Laura Milena Forero Junco[1,**], Jacob Bruun Jørgensen[1], Tue Kjærgaard Nielsen[1], Morten Arendt Rasmussen[3], Witold Kot[1,*] and, Lars Hestbjerg Hansen[1,*]**

[1] Department of Plant and Environmental Sciences, University of Copenhagen, 1871 Frederiksberg C, Denmark; laura.junco@plen.ku.dk (L.M.F.J.), jacobbruun@protonmail.com (J.B.J.) and tkn@plen.ku.dk (T.K.N.)
[2] Department of Environmental Science, Aarhus University, 4000 Roskilde, Denmark; Ksala@envs.au.dk (K.W.S.A.)
[3] Department of Food Science, University of Copenhagen, 1958 Frederiksberg C, Denmark; M.A.R mortenr@food.ku.dk
[4] COPSAC, Copenhagen Prospective Studies on Asthma in Childhood, Herlev and Gentofte Hospital, University of Copenhagen, 2820 Gentofte, Denmark
* Correspondence: L.H.H. lhha@plen.ku.dk and W. K. wk@plen.ku.dk
** These authors contributed equally to the work presented

Supplementary material

All supplementary material for the article ''Spiked viromes reveal host-specific lytic phages uncultured by plaque assay''. These include:

Figure S1: Overview of the experimental setup, plaque assay and key findings
Table S2: Overview of DNA amount, quality, and reads after quality control (QC)
Table S3: Virsorter2 results on contigs
Table S4: Virsorter2 results on bp
Table S5: CheckV result on individual assemblies
Figure S6: Viral OTU composition
Figure S7: Accumulative abundance of all contigs in the three sample types
Table S8: Observed vOTUs in each of the virome samples
Table S9: Read mapping coverage of plaque-forming phages from organic waste sample (separate excel sheet)
Table S10: CRISPR hits on host taxonomy at species level
Table S11: Putative phage contigs that has a Log2FC above 1 or below – 1
Table S12: Putative phage contigs that has a Log2FC between -1 and 1 without a significant abundance

Figure S1

Figure S1: Overview of the experimental setup, sequencing platforms, and analysis. **Plaque assay**: Five single phages were isolated with either direct plating (Hovsa and GhostToast), PEG purification followed by direct plating (DrKristoffer and OtownIsak), or enrichment overnight similar to the incubation for DC3000 + and DC3000 – (SummerBoyErik). **Virome**: Visualization of the three types of viromes, DC3000 +, DC3000 – and Baseline. Incubation time for DC3000 + and DC3000 – was 8 hours at room temperature. **Key findings:** A total of 101,068 putative viral contigs was identified from all merged reads, and clustered into 35,070 viral operational taxonomical units referred to as viral populations (vOTUs), where 3,515 were >10 kb. We identified three of the five phages in the viromes, but observed that OtownIsak or Hovsa was the most enriched phage when incubated with DC3000 as they belonged to the same vOTU. *Pseudomonas* phages were confirmed to be significantly enriched when incubated with DC3000. Created with BioRender.com and restructured in Adobe Illustrator.

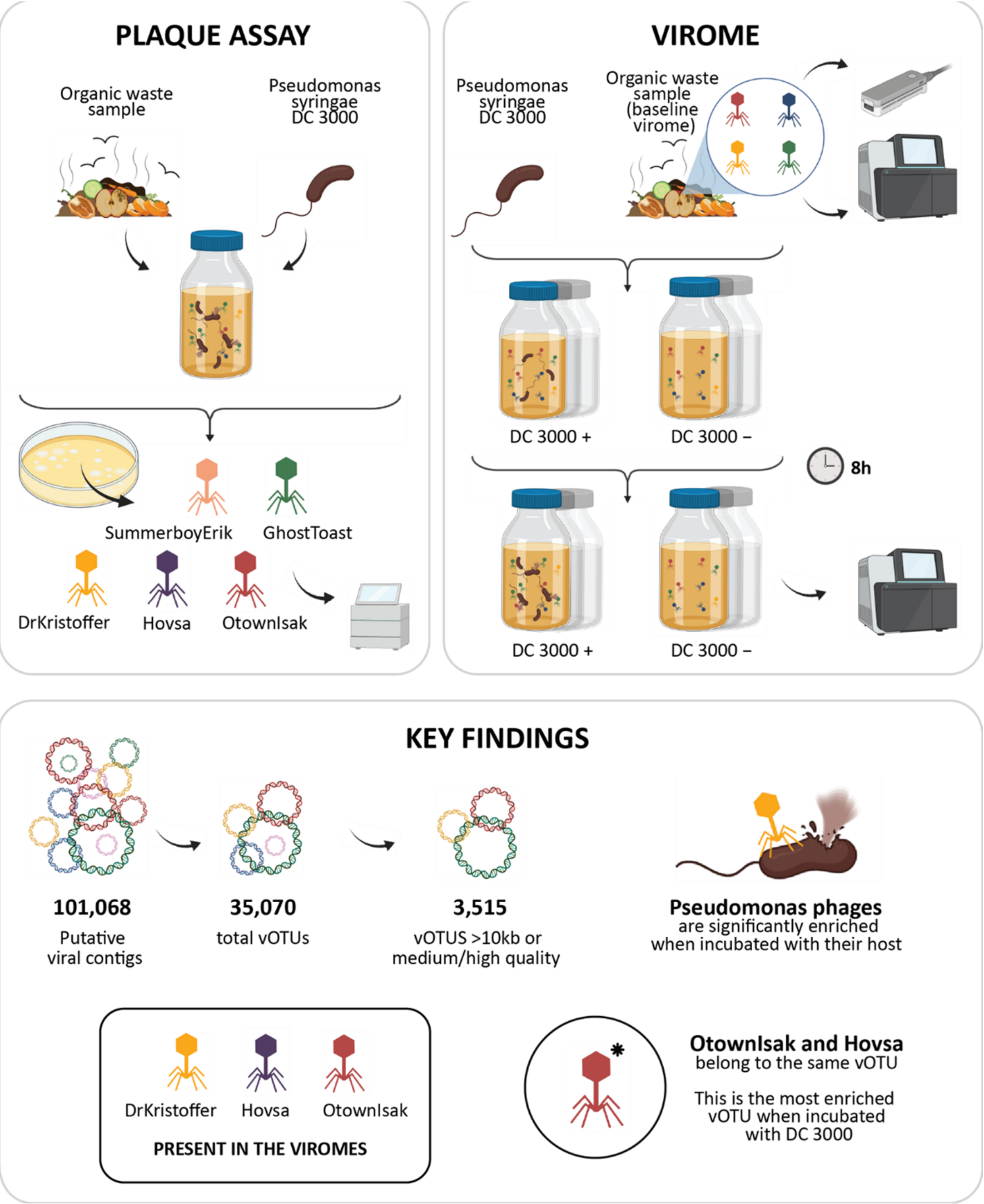

Table S2

Table S2: Overview of DNA amount, quality, and reads after quality control (QC) for each of the seven viromes used for the abundance analysis. N = Nanopore reads, P = paired Illumina reads, and UP = unpaired Illumina reads.

Virome	Total DNA (ng)	Nanodrop (260/280) / (260/230)	Post QC reads
Baseline	2,520	(1.2) / (0.7)	N: 1251909, P: 9500926, UP: 453784
DC3000+ A	543	(1.9) / (2.1)	P: 3641173, UP: 275037
DC3000+ B	474	(1.8) / (2.1)	P: 4328342, UP: 350347
DC3000+ C	597	(1.9) / (2.0)	P: 4558370, UP: 1861554
DC3000- A	4.76	(0.3) / (0.2)	P: 5227108, UP: 333565
DC3000- B	25.6	(0.4) / (0.3)	P: 4970642, UP: 438710
DC3000- C	24.4	(0.4) / (0.3)	P: 5596835, UP: 408592

Table S3

Table S3: Virsorter2 results on contigs. Data of total contigs, as well as how much was identified putative viral and putative non-viral by VirSorter2.

Assembly	No. of putative viral contigs	No. of putative non-viral contigs	Total no. of contigs	% putative viral contigs	% putative non-viral contigs
Baseline metaSPAdes (Assembly 1)	11,867	3,915	15,782	75.2	24.8
DC3000 + metaSPAdes (Assembly 2)	9,481	2,690	12,171	77.9	22.1
DC3000 + A metaSPAdes (Assembly 3)	3,738	883	4,621	80.9	19.1
DC3000 + B metaSPAdes (Assembly 4)	4,155	1,091	5,246	79,2	20.8
DC3000 + C metaSPAdes (Assembly 5)	2,818	686	3,504	80.4	19.6
DC3000 – metaSPAdes (Assembly 6)	16,925	5,332	22,257	76.0	24.0
DC3000 – A metaSPAdes (Assembly 7)	6,796	1,904	8,700	78.1	21.9
DC3000 – B metaSPAdes (Assembly 8)	6,389	1,860	8,249	77.5	22.5
DC3000 – C metaSPAdes (Assembly 9)	6,647	1,865	8,512	78.1	21.9
All samples hybrid metaSPAdes (Assembly 10)	30,068	9,666	39,734	75.7	24.3
Baseline Canu (Assembly 11)	2,184	113	2,297	95.1	4.9
Baseline Canu (Assembly 12)	2,184	113	2,297	95.1	4.9

Table S4

Table S4: Virsorter2 results on bp. Data of total bp, as well as how much was identified putative viral and putative non-viral by VirSorter2

Assembly	No. of putative viral bp	No. of putative non-viral bp	Total no. of bp	% putative viral bp	% putative non-viral bp
Baseline metaSPAdes (Assembly 1)	29,835,217	5,643,379	35,478,596	84.1	15.9
DC3000 + metaSPAdes (Assembly 2)	21,504,440	3,661,585	25,166,025	85.5	14.5
DC3000 + A metaSPAdes (Assembly 3)	9,004,304	1,196,940	10,201,244	88.3	11.7
DC3000 + B metaSPAdes (Assembly 4)	9,500,589	1,466,266	10,966,855	86.6	13.4
DC3000 + C metaSPAdes (Assembly 5)	6,364,929	926,243	7,291,172	87.3	12.7
DC3000 – metaSPAdes (Assembly 6)	39,008,458	7,481,007	46,489,465	83.9	16.1
DC3000 – A metaSPAdes (Assembly 7)	15,563,712	2,596,593	18,160,305	85.7	14.3
DC3000 – B metaSPAdes (Assembly 8)	14,712,604	2,586,311	17,298,915	85.0	15.0
DC3000 – C metaSPAdes (Assembly 9)	15,557,533	2,593,590	18,151,123	85.7	14.3
All samples hybrid metaSPAdes (Assembly 10)	86,640,254	14,128,443	100,768,697	86.0	14.0
Baseline Canu (Assembly 11)	27,445,358	314,256	27,759,614	98.9	1.1
Baseline Canu (Assembly 12)	27,436,635	313,952	27,750,587	98.9	1.1

Table S5

Table S5: CheckV result on individual assemblies. Distribution of contig quality identification by CheckV in regards to phage genome completeness for each of the 11 assemblies

Assembly	Complete/high	Medium	Low	Not determined	Total vOTUs
Baseline metaSPAdes (Assembly 1)	29	51	12,274	2,434	14,788
DC3000 + metaSPAdes (Assembly 2)	17	31	9,865	1,532	11,445
DC3000 + A metaSPAdes (Assembly 3)	6	19	3,805	492	4,916
DC3000 + B metaSPAdes (Assembly 4)	7	20	4,346	582	4,955
DC3000 + C metaSPAdes (Assembly 5)	5	11	2,941	385	3,342
DC3000 – metaSPAdes (Assembly 6)	26	52	17,504	3,204	20,786
DC3000 – A metaSPAdes (Assembly 7)	15	19	7,060	1,048	8,142
DC3000 – B metaSPAdes (Assembly 8)	9	28	6,808	899	7,744
DC3000 – C metaSPAdes (Assembly 9)	12	23	6,912	1,044	7,991
All samples hybrid metaSPAdes (Assembly 10)	132	196	29,751	6,401	36,480
Baseline Canu (Assembly 11)	116	171	1,595	122	2,004

Table S6

Table S6: Viral OTU composition of the population present in the three replicates of virome DC3000 + and DC3000 − and the baseline virome. **A** Hierarchical clustering of vOTUs based on Jaccard distances. **B** Pairwise Bray-Curtis distances. **C** PCoA plot. **D** Comparison of treatment – incubation of OW in LB media at room temperature for 8 hours with or without an overnight culture of DC3000. ANOSIM test statistic = 0.42 (p-value = 0.106).

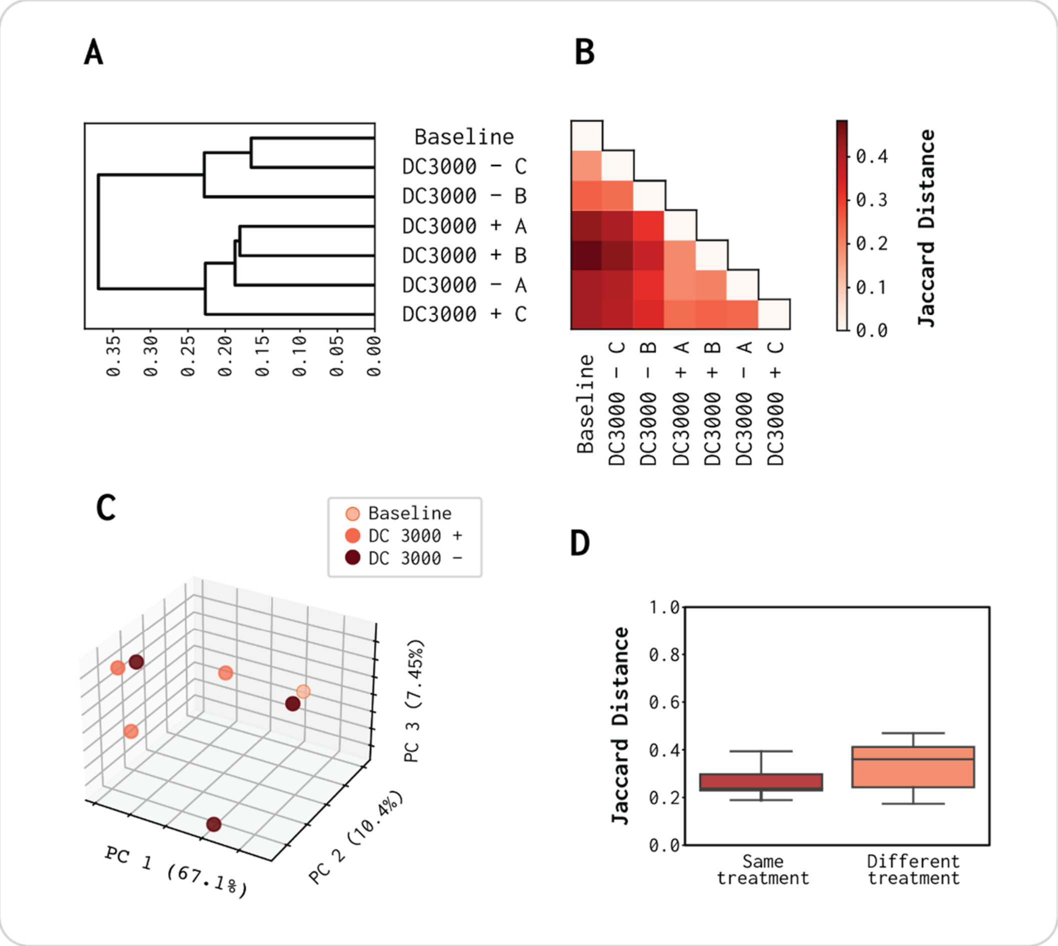

Figure S7

Figure S7: Accumulative abundance of all contigs in the three virome types.

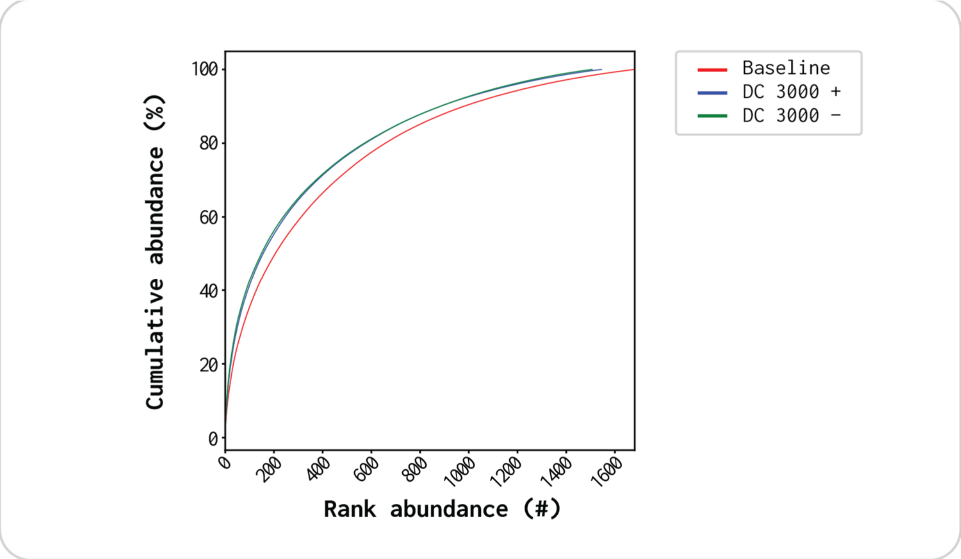

Table S8

Table S8: Observed vOTUs in each of the virome samples used for Figure 4 in the main text.

Sample	Observed vOTUs
Baseline	1387
DC3000 + A	1547
DC3000 + B	1450
DC3000 + C	1316
DC3000 – A	1508
DC3000 – B	1500
DC3000 – C	1417

Table S10

Table S10: CRISPR hits on host taxonomy at species level. Out of the 3,515 viral populations, 1164 had hits to a bacterial CRISPR spacer from the Shmakov database. A total of 806 of those hits could be assigned to a host at the species level. Below are the genera with ≥ 5 assigned viral populations.

Genus	Count	Genus	Count
Lactobacillus	209	*Bifidobacterium*	10
Pseudomonas	47	*Bacillus*	9
Xanthomonas	18	*Salmonella*	7
Dickeya	18	*Aeromonas*	7
Acinetobacter	17	*Klebsiella*	7
Pediococcus	14	*Delftia*	6
Streptococcus	13	*Yersinia*	6
Escherichia	12	*Pluralibacter*	5
Serratia	11	*Porphyromonas*	5
Acetobacter	11	*Bacteroides*	5

Table S11

Table S11: Putative phage contigs that has a Log2FC above 1 (Green dots) or below – 1 (Red dots) (Figure 5A). Contigs were blasted using megablast. Denoted below are the top viral hit with lowest E-value and highest query coverage.

Contig	Size (Bp)	> 1: Log2FC > 1<br>< -1: Log2FC < -1	Blast hit (Accession no.)	Query coverage	Per. identity	E-value
tig00010143	20829	> 1	Lactobacillus phage iLp1308 (KR905070.1)	7%	77.16%	0.0
NODE_45	50899	> 1	Siphoviridae NHS-Seq1 (MH029512.1)	32%	80%	0.0
tig00000588	18290	> 1	Lactobacillus phage 3-SAC12 (MK504442.1)	3%	91.75%	2×10^{-161}
NODE_3	59207	> 1	Enterobacteria phage CAjan (KP064094.1)	100%	100%	0.0
NODE_45	10380	> 1	Lactococcus phage 56301 (NC_049405.1)	45%	85.77%	0.0
NODE_220	5166	> 1	Lactobacillus phage AQ113 (HE956704.1)	8%	84.40%	3×10^{-119}
NODE_2743	5420	> 1	Leuconostoc phage CHB (KX578043.1)	8%	67.40%	3×10^{-29}
tig00001464	9726	< -1	Lactobacillus phage Lenus (NC_047897.1)	89%	97.35%	0.0
NODE_830	5136	< -1	Paramecium bursaria Chlerella virus CVM-1 (JX997163.1)	1%	75.29%	0.003
NODE_690	6178	< -1	Lactobacillus phage 3-521 (NC_048753.1)	100%	97.28%	0.0
NODE_2693	5480	< -1	Lactobacillus phage 3-521 (NC_048753.1)	93%	97.70%	0.0
NODE_132	40054	< -1	Uncultured Caudivirales phage (LR796317.1)	1%	68.32%	1×10^{-37}
tig00000648	32820	< -1	Lactobacillus phage LR2 (MH837543.1)	5%	67.97%	3×10^{-64}
tig00000040	44196	< -1	Escherichia phage C130_2 (NC_048067.1)	2%	76.66%	4×10^{-127}
NODE_852	11518	< -1	Phage apr34_1789 (MK415401.1)	0%	80.21%	2×10^{-11}
tig00000828	11199	< -1	Proteus phage vB_PmiP_RS8pmA (MG575419.1)	88%	93.04%	0.0
NODE_830	5580	< -1	*Lactococcus lactis. Subs. Lactis strain G423* (CP024958.1)	1%	80%	0.047
NODE_214	32484	< -1	Proteus phage PM75 (NC_027363.1)	61%	92.01%	0.0

Table S12

Table S12: Putative phage contigs that has a Log2FC between -1 and 1 without a significant abundance (Figure 5A). Contigs were blasted using megablast. Denoted below are the top viral hit with lowest E-value and highest query coverage.

Contig	Size (Bp)	0-1: Log2FC 0-1<br>-1-0: Log2FC -1-0	Blast hit (accession no.)	Query coverage	Per. identity	E-value

NODE_364	6226	0-1	Proteus phage vB_PmiP_RS51pmB (MG575421.1)	6%	79.59%	5×10^{-44}
NODE_209	8610	0-1	Bacteriophage sp. Isolate 179 (MN855830.1)	54%	84.73%	0.0
NODE_445	5408	0-1	Lactobacillus phage ATCCB (MK504445.1)	21%	76.79%	1×10^{-174}
tig00001258	5764	0-1	Lactobacillus phage AQ113 (HE956704.1)	16%	72.53%	1×10^{-154}
NODE_2231	6180	0-1	Myoviridae sp. ctThM1 (MN582070.1)	9%	72.78%	9×10^{-50}
NODE_507	5015	0-1	Bacteriophage sp. Isolate 103 (MN855801.1)	3%	73.96%	1×10^{-15}
NODE_165	6157	0-1	Bacteriophage sp. Isolate 32 (MN855637.1)	0%	88.89%	4×10^{-05}
NODE_281	9947	-1-0	Xylella phage Usme (LR743523.1)	1%	75.94%	1×10^{-46}
NODE_244	10727	-1-0	Salmonella phage assan (MT074440.1)	20%	69.49%	3×10^{-97}
tig00000616	17334	-1-0	Lactobacillus phage CL2 (KR905067.1)	5%	83.54%	0.0
tig00010057	16105	-1-0	Lactobacillus phage LBR48 (GU967410.1)	8%	92.67%	0.0
NODE_966	5065	-1-0	Bacteriophage sp. isolate 230 (MN855846.1)	41%	83.45%	0.0
NODE_1644	7563	-1-0	Lactobacillus phage ATCC 8014-B2 (NC_047739.1)	22%	80.31%	0.0
NODE_2505	5716	-1-0	Lactobacillus phage ATCC 8014-B2 (NC_047739.1)	84%	85.48%	0.0
